# Development and Validation of a High-Throughput Mass Spectrometry Based Urine Metabolomic Test for the Detection of Colonic Adenomatous Polyps

**DOI:** 10.3390/metabo7030032

**Published:** 2017-06-22

**Authors:** Lu Deng, David Chang, Rae R. Foshaug, Roman Eisner, Victor K. Tso, David S. Wishart, Richard N. Fedorak

**Affiliations:** 1 Metabolomic Technologies Inc., Edmonton, AB T6N 1G1, Canada; david.chang@metabolomictechnologies.ca (D.C.); rae.foshaug@metabolomictechnologies.ca (R.R.F.); reisner@ualberta.ca (R.E.); vtso@ualberta.ca (V.K.T.); 2 Department of Biological Sciences, University of Alberta, Edmonton, AB T6G 2E9, Canada; david.wishart@ualberta.ca; 3 Department of Medicine, University of Alberta, Edmonton, AB T6G 2G3, Canada; richard.fedorak@ualberta.ca

**Keywords:** colorectal cancer, adenomatous polyps, metabolomics, metabolite, urine, diagnostic test, MS, NMR

## Abstract

*Background:* Colorectal cancer is one of the leading causes of cancer deaths worldwide. The detection and removal of the precursors to colorectal cancer, adenomatous polyps, is the key for screening. The aim of this study was to develop a clinically scalable (high throughput, low cost, and high sensitivity) mass spectrometry (MS)-based urine metabolomic test for the detection of adenomatous polyps. *Methods*: Prospective urine and stool samples were collected from 685 participants enrolled in a colorectal cancer screening program to undergo colonoscopy examination. Statistical analysis was performed on 69 urine metabolites measured by one-dimensional nuclear magnetic resonance spectroscopy to identify key metabolites. A targeted MS assay was then developed to quantify the key metabolites in urine. A MS-based urine metabolomic diagnostic test for adenomatous polyps was established using 67% samples (un-blinded training set) and validated using the remaining 33% samples (blinded testing set). *Results*: The MS-based urine metabolomic test identifies patients with colonic adenomatous polyps with an AUC of 0.692, outperforming the NMR based predictor with an AUC of 0.670. *Conclusion*: Here we describe a clinically scalable MS-based urine metabolomic test that identifies patients with adenomatous polyps at a higher level of sensitivity (86%) over current fecal-based tests (<18%).

## 1. Introduction

Colorectal cancer (CRC) is a major public health concern. Globally, it is ranked as the third most frequent form of cancer with an age-standardized incidence rate of 17.2 per 100,000 population making it responsible for almost 8.5% of all deaths due to cancer [1]. CRC is also the third leading cause of cancer-related deaths in the Western world [2]. CRC is largely preventable through population-based and individual-based screening programs that aim to detect adenomatous polyps, the precursor to CRC, as more than 95% of CRC develops from adenomatous polyps [3]. Colonoscopy is the gold standard for identifying both CRC and polyps. In an ideal world, every at-risk subject would receive a colonoscopy, since the cost of a colonoscopy (~CDN $1200) is significantly lower for the health industry than the expected cost of treating the possible colon cancer (~CDN $20,000) [4]. However, the cost of colonoscopy and its associated morbidity and mortality precludes it as a cost-effective population-based screening test. Currently, non-invasive, fecal-based testing is the foundation to most screening programs. Fecal-based testing is only used to determine which individuals should receive a colonoscopy [5], the definitive test for identifying and removing adenomatous polyps. Unfortunately, there are several factors that limit the effectiveness of the fecal-based testing as a screening method. The first is that relatively few individuals complete the standard fecal-based testing, including those known to be at above-average risk for CRC [6]. Second, the fecal-based diagnostic tests have low sensitivity [7]. The guaiac-based fecal test, which tests for hemoglobin, has a sensitivity of approximately 3% for detecting any adenoma and 10–30% for detecting advanced (>10 mm) adenomatous polyps [8]. Newer fecal immunochemical tests, which use antibodies to hemoglobin, have reported sensitivities of 13–26% for any adenomatous polyps [9] and 20–67% for advanced adenomatous polyps [10]. Third, the interpretation of these fecal-based test is subjective as the result is a colorimetric change, which means it can be difficult to determine whether the test is truly positive or not. A better, more accurate, more patient compliant and much less expensive “Colonoscopy Predictor” (i.e., a test that can accurately predict whether a patient has an adenomatous polyp and should receive a colonoscopy) would serve as the ideal population-based CRC screening test.

Metabolomics is a new “omics” science that focuses on characterizing low molecular weight compounds generated by metabolism. Metabolomics offers a dynamic portrait of the metabolic status of living systems [11]. There are more than 40,000 metabolites known to be in the human body [12], and their specific concentrations provide a snapshot of an individual’s current state of health. Urine has long been a “favored” biofluid among metabolomics researchers. It is sterile, easy-to-obtain in large volumes, largely free from interfering proteins or lipids and chemically complex. More than 2650 metabolites have been identified, to date, in human urine samples [13]. These include amino acids, nucleic acids, carbohydrates, organic acids, vitamins, lipids, minerals, food additives, drugs, toxins, pollutants, and other chemicals (with a molecular weight < 2000 Da) that humans ingest, metabolize, catabolize, or come into contact with [12]. With respect to adenomatous polyps and CRC, metabolomics has demonstrated the capacity to detect not only dysplastic cellular changes of the human mucosa [14], but also changes in the intestinal microflora [15]. To date, one systematic review [16] and ten pilot studies have examined how metabolomics can be used to identify CRC but only two studies explored metabolomics for detection of adenomatous polyps [17,18]. Haili Wang and Richard Fedorak, et al. have recently developed a metabolomic based urine test for the detection of adenomatous polyps, the precursor to CRC [18]. Through the metabolic profiling by one-dimensional nuclear magnetic resonance spectroscopy (NMR) of nearly 1000 urine samples at the University of Alberta (Edmonton, AB, Canada), 14 metabolites were found to be significant metabolites to separate individuals with polyps from those without polyps. A prototypic diagnostic test for the detection of adenomatous polyps was established using the concentration of the 14 urinary metabolites along with clinical features (e.g., age, sex, smoking history). Using the colonoscopy results as a gold standard, the NMR-based urine metabolomic test was able to detect colonic adenomatous polyps with a sensitivity of 88.9%, a specificity of 50.2%, and an area under curve (AUC) of 0.7524 [18]. This urine metabolomic diagnostic test was shown to have a higher sensitivity than the Fecal Guaiac HemII, Fecal Immune ICT, and Fecal Immune MagSt tests, which are currently in use. However, further refinement and development was needed to bring this prototypic urine-based metabolomic test to clinical use for the following reasons: (1) NMR is a relatively expensive and large piece of equipment. It requires a high level of expertise to maintain and operate. Currently, it is mostly used in research domain, but not readily available for clinical usage. So, there is a need here to transfer the test from NMR platform to a cheaper and readily available analytical platform in clinical settings for easy adoption; (2) For the prototypic NMR test, it takes 6 min per sample to for instrument running and 20 min per sample for metabolite quantification. We aim to develop a metabolomic based urine test that would be suitable for a population-based CRC screening tool. High throughput and low cost are key factors here.

The aim of this study was to develop and validate a clinically scalable (high throughput, low cost, and high sensitivity) diagnostic test for the detection of adenomatous polyps, which would be suitable for population-based CRC screening. This was accomplished by the development of a targeted liquid chromatograph (LC)-MS/MS method for the quantification of key metabolites in 685 urine samples. The metabolites identified and quantified by MS were then compared with those identified and quantified by NMR. Using the MS-derived metabolite concentration data we built a predictor that determines whether a patient requires a colonoscopy for adenomatous polyp removal. The AUC was calculated for the newly developed MS-based test and compared to the NMR test. The sensitivity and specificity of this high-throughput MS-based urine diagnostic test was also compared with commercially available fecal-based tests.

## 2. Materials and Methods

### 2.1. Study Participants and Sample Collection

This study used 685 urine samples (collected from April 2008 to October 2009) that were obtained as part of a regional colon cancer screening program, in Edmonton, Canada (SCOPE^®^, Stop Colorectal Cancer through Prevention and Education) [7,9,18,19]. Study participants of average CRC risk (50–75 years 50–75 years of age and no personal or first-degree family history of CRC or polyps) or increased CRC risk ((40–75 years of age with a personal or first-degree family history of CRC or polyps) were recruited. On day of entry, participants provided informed consent, a midstream urine sample, and completed a demographic survey. No dietary collection or activity modification was required before the urine collection. Within 1 week of providing the urine sample, all participants provided a fecal sample to undergo fecal occult blood testing using three commercially available tests. The Hemoccult II (Beckman Coulter, Mississauga, ON, Canada) test (non-rehydrated) was positive if at least one test window displayed a blue color within 60 s of developer. The Hemoccult ICT (Beckman Coulter) was positive if a pink line appeared in the test area within 5 min of buffer. The MagStream HemSp/HT (Fujirebio Diagnostics, Malvern, PA, USA) was positive at a level of 467 mg hemoglobin/g stool. Colonoscopy was performed 2–6 weeks after the urine and stool collections as the reference standard. Participants were excluded if they had findings of colonic or ileal disease at the time of colonoscopy. All subjects gave their informed consent for inclusion before they participated in the study. The study was conducted in accordance with the Declaration of Helsinki, and the protocol was approved by the Health Research Ethics Board at the University of Alberta (Project identification number: Pro00000514). The www.ClinicalTrials.gov identifier is NCT01486745.

### 2.2. NMR Analysis

This study used NMR spectra of 685 urine samples previously obtained [18]. NMR spectra were collected on a 600 MHz NMR spectrometer equipped with a VNMRS two-channel console (Varian Inc., Palo Alto, CA, USA). Additional NMR acquisition details can be found elsewhere [18]. The metabolite quantification was first performed in 2010 using the targeted profiling techniques of Chenomx NMR Suite v7.7 (Chenomx Inc., Edmonton, AB, Canada) [18] and then re-profiled in 2013 using the same protocol but with different operators to investigate the consistency of metabolite determination from NMR spectra over time and between operators [19]. For this study, we used the metabolite quantification results generated in 2013.

### 2.3. Mass Spectrometry Analysis

A targeted LC-MS/MS method was developed to quantify three key metabolites (succinic acid, ascorbic acid, and carnitine identified from the previous NMR studies) in urine samples using multiple reaction monitoring (MRM) on an Agilent 1290 UHPLC/AB Sciex 4000 Qtrap system.

### 2.4. Standards

Succinic acid (BioXtra, ≥99.0%), L-Ascorbic acid (BioXtra, ≥99.0%, crystalline), and L-Carnitine (synthetic, ≥98%) were purchased from Sigma-Aldrich (Oakville, ON, Canada). Succinic acid (D4, 98%), L-Ascorbic acid (1-^13^C, 99%), and L-Carnitine (Trimethyl-D9, 98%) were purchased from Cambridge Isotope Laboratories (Tewksbury, MA, USA). Stock solutions of individual compounds were made by dissolving proper amounts of each standard in MS grade water. Targeted NMR analysis on stock solutions of unlabeled standards were also performed to confirm the standard quality. Calibrant solutions (Cal1-Cal8) at concentrations of 5 µM, 10 µM, 100 µM, 200 µM, 400 µM, 600 µM, 800 µM, and 1000 µM were prepared by mixing the stock solutions of unlabeled succinic acid, ascorbic acid, and carnitine in water. An internal standard solution (ISTD) with 100 µM of succinic acid-D4, 200 µM of ascorbic acid-^13^C, and 100 µM of Carnitine-D9 was prepared by mixing the stock solutions of isotopic labeled internal standards in MS grade water. Calibrant solutions and the internal standard solution were aliquoted and stored at −80 °C until used.

### 2.5. Sample Processing

All urine samples were processed in a 96-well plate format. Each plate consisted of 1 blank solution, 1 ISTD, 8 calibration solutions (Cal1-Cal8), 6 quality control (QC) samples and 80 urine samples. Laboratory-generated pooled urine samples from 6 healthy individuals served as the QC samples. A simple approach of dilution and filtration was used for sample preparation. Urine samples and calibration solutions were left to thaw on ice and centrifuged at 10,000 *g* for 3 min. 10 µL of each urine supernatant or calibration solution was added to the corresponding well in each plate. 10 µL of the ISTD was added to each well on the plate, except the blank well (A1). This was done to account for matrix effects and to facilitate absolute quantification of the metabolite concentrations. The mixture was extracted with 200 µL of extraction solvent (water with 10 mM ammonium formate, pH3) and filtered through a 0.45 µm member filter before LC-MS injection.

### 2.6. LC-MS Analysis

LC-MS spectra were acquired on an AB Sciex 4000 Qtrap paired to an Agilent UHPLC 1290. An isocratic LC separation of the targeted metabolites (succinic acid, ascorbic acid, and carnitine) was performed using a Waters ACQUITY UPLC BEH C18 column (2.1 mm × 150 mm, 1.7 µm) with 95:5 water:acetonitrile (10 mM Ammonium formate, pH3) as mobile phase and a flowrate of 0.3 mL/min. The injection volume was 5 µL and the overall LC run time was 3 min. MRM detection was under optimal parameters for each of the analytes. Metabolite quantification was achieved using the AB Sciex Analyst^®^ software version 1.6.2. During quantification, each metabolite was identified using the internal standard and quantified using the established calibration curve.

### 2.7. Statistical Analysis

**Figure 1:**
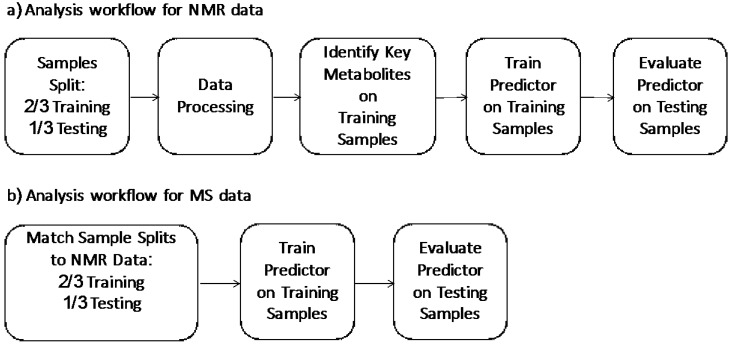
(**a**) Analysis workflow for NMR data; (**b**) analysis workflow for MS data.

In the development of the test, we wanted to evaluate the performance in the strictest way possible and in real world situations, where the test is required to predict labels for new un-labeled instances. As such we followed the standard machine learning methodology [20,21] of using an external data set to evaluate our predictor. Our general analysis workflow is outlined in Figure 1. Figure 1a outlines our analysis workflow on the NMR data. Initially, we split the NMR dataset into training data (2/3, *n* = 457) and testing data (1/3, *n* = 228). These two data sets were balanced for age, sex, and class distribution. This training data was then used to identify important metabolites. These identified key metabolites were used in conjunction with our clinical features (age, sex, smoking status) to train a NMR predictor. This was then evaluated on the testing data set. Figure 1b outlines our analysis workflow on MS predictor. We matched the training and testing splits of the samples to the NMR splits in the NMR analysis. A MS predictor was developed using identified key metabolites by LC-MS in conjunction with three clinical features (age, sex, smoking status) of the training set and further validated by the testing set.

For both the NMR and MS predictors, machine learning algorithms were used to generate an optimal diagnostic predictor from the measured concentration data. All code was written in R (version 3.2.0). SVM, SVMRBF, RF, LASSO, NB, C5.0, PLSDA, SVM-Tune, SVM-Cost, Logistic, and KNN [22] were evaluated and best prediction result from LASSO was reported. For LASSO [23], we used the glmnet R library (version 2.0-2). Optimization of the lambda parameter was done using the cv.glmnet function in the *glmnet* package. For other evaluated algorithms, details can be found elsewhere [7]. Both the NMR and the MS metabolite quantification results were log-transformed. Metabolite concentrations that were below the lower limit of detection (LLOD) were replaced by half of the limit of detection for statistical analysis.

## 3. Results

### 3.1. Participant Characteristics

Of the 685 participants selected for this study, colonoscopy results partition the subjects into one of four outcomes (from least severe to most severe): subjects that have (i) no abnormality (Normal, *n* = 446); (ii) only hyperplastic polyps (Hyperplastic, *n* = 84); (iii) adenomatous polyps (Adenoma, *n* = 154); or (iv) colorectal cancer (CRC, *n* = 1); see Table 1. As the goal of this study is to develop a test that can be used for population based CRC screening, subjects with no abnormality and only hyperplastic polyps were classified as “Normal”, while subjects with adenomatous polyps and CRC were classified as “Polyp”. The characteristics of three important clinical features, age, sex, and smoking history are also shown in Table 1.

### 3.2. Key Metabolite Identification

The metabolite quantification by NMR on the 685 urine samples was first performed in 2010 using the targeted profiling techniques of Chenomx NMR Suite v7.7 (Chenomx, Inc., Edmonton, AB, Canada) [18]. In a later consistency study, re-quantification was carried out using the same NMR spectra and same protocol, but by different operators and at different time points [19]. The authors compared the analyzed concentration data among operators and time. Interestingly, the consistency of the analyzed NMR results is dependent on the metabolite identity with the more difficult to profile metabolites being more inconsistent. The spectral analysis team categorized each of the identified 69 metabolites into one of four consistency groups: Excellent, Good, Fair, and Poor. To ensure greater consistency and to remove any batch effects, we removed 13 of the 69 metabolites that were rated “Poor”. Further, any metabolites that were zero for more than 20% of the sample set were not considered (e.g., 3-hydroxymandelic acid). Important features that could distinguish those with adenomatous polyps from those without, were identified using the reliable metabolite abundance information. The NMR dataset were split into training data (2/3, *n* = 457) and testing data (1/3, *n* = 228). These two data sets were balanced for age, sex, and class distribution. This training data was then used to identify important metabolites. After further data processing (scaling and normalization), metabolites were ranked using their *p*-value (via the Wilcoxon signed-rank test), as listed in Table 2. The three key metabolites that had a *p*-value less than 0.05 were: succinic acid, ascorbic acid, and carnitine.

## 4. LC-MS Measurements

*LC-MS acquisition.* MS parameter optimization was performed on an AB Sciex 4000 Qtrap for each metabolite using a standard solution of 5 µM of the compound in a 1:1 water:acetonitrile buffer with 0.1% formic acid. For each compound, two of the most abundant MRM pairs were chosen and the corresponding MS parameters, such as De-clustering Potential (DP), Collision Energy (CE) and Collision Cell Exit (CXP) were optimized. All of the MS parameters are summarized in Table S1 (Supplemental Data). Succinic acid and ascorbic acid were monitored in the negative mode, while carnitine was monitored in the positive mode. MRM pair 1 (e.g., succinic acid 1) was used for quantification analysis, MRM pair 2 (e.g., succinic acid 2) was monitored for identification. A representative LC-MS chromatograph of one of the calibration solutions (Calibrant 6) is shown in Figure S1 (Supplemental Data) to illustrate the level of LC separation achieved with this protocol. Succinic acid and ascorbic acid are clearly baseline separated.

*LC-MS assessment.* 685 urine samples were randomized and run through the developed LC-MS method with a 96 well plate format. Each plate contains 1 blank, 1 ISTD, 8 Calibrants, 6 QCs (laboratory generated pooled urine samples), and 80 urine samples from the study participants. A representative plate map is shown in Figure S2 (Supplemental Data). A total of 9 plates were run.

Method assessment and validation was performed according to Clinical and Laboratory Standards Institute (CLSI) C62A guidelines [24]. LC-MS analysis was done on non-spiked urine, spiked urine, and post-spiked urine samples in triplicate. Extraction recoveries and accuracies were calculated for each metabolite and summarized in Table S2 (Supplemental Data). All metabolites were within the range of 90–110%. For each plate, a set of calibration curves was generated and used. Linear regression coefficients (R^2^) for the 3 measured metabolites were > 0.99 for all plates. 6 QC samples were put into each plate to access the coefficient of variation (CV%) with the plate and across the 13 different plates. The CV% of the QC samples for each metabolite within each plate was calculated as ratio of the standard deviation to the mean value, summarized in the Table S3 (Supplemental Data). Notably, the CV% for each metabolite with the plate was < 15%. The average concentration of the measured metabolite in QC samples were also listed in Table S3 (Supplemental Data). The concentrations of succinic acid, ascorbic acid, and carnitine were consistent across the 9 plates within acceptable ranges.

### 4.1. Development and Validation of the MS Based Test

**Figure 2:**
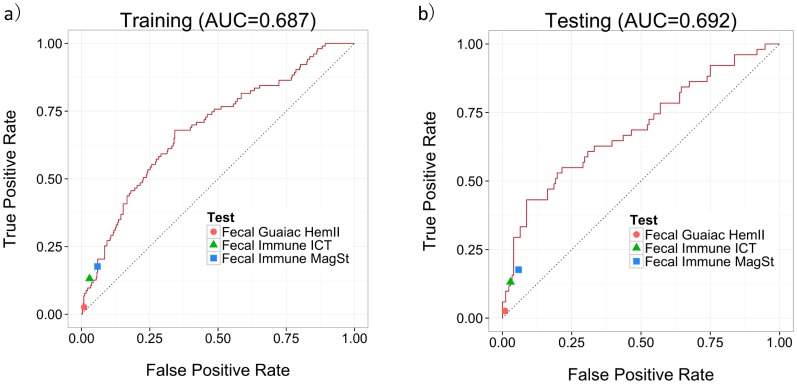
Performance of MS-based predictor using 3 metabolites and 3 clinical features on (**a**) the training data; and (**b**) the testing data, including the performance of the fecal based tests.

Since we were focused on how well our predictor could predict labels for new un-labeled instances, we followed the standard machine learning methodology [20,21] of using an external data set to evaluate our predictor. The dataset was divided into 2/3 training data and 1/3 testing data. These two datasets were balanced for age, sex, and class distribution. The MS quantifications were log-transformed and were used in conjunction with three clinical features (age, sex, and smoking status) along with a label (specifically, “Polyp” or “Normal”) to train a predictor using the LASSO algorithm. The LLOQ for the LC-MS/MS assay was set to be the lowest calibrant point at *5* μM. Metabolite concentrations that were below the LLOQ were replaced by half of the lower limit of detection to facilitate further statistical analysis. The trained predictor was then evaluated on the testing data set using sensitivity, specificity and AUC of the Receiver Operating Characteristic (ROC) curve. Figure 2a,b show the ROC curves of our predictor’s performance on the training and testing data, respectively. An AUC of 0.687 was achieved on the training set and an AUC of 0.692 was achieved on the testing set.

One of the advantages of our algorithm is that the prediction threshold is adjustable, allowing one to vary the tradeoff between sensitivity and specificity. To examine how confidently we can specify the predictive performance of the MS-based metabolomics test to given physician recommendations, we picked several prediction thresholds on the training data results (along the ROC curve), and evaluated on the testing data using the same prediction threshold. The results are summarized in Table 3 at several intuitive thresholds: sensitivities at 70%, 80%, and 90%, and specificities at 70%, 80%, and 90%. The results show that this protocol for picking a threshold generalizes well to the testing set. This is probably due to the nature of the LASSO linear predictor. For more complex predictors, such as Random Forests, this threshold selection does not generalize well.

We also ran permutation tests [25] to determine whether the MS-based predictor was indeed finding useful patterns. This involved randomizing the labels in the training set, then running the training/testing workflow. The result of this analysis is expected to be worse than the performance of our predictor, as the labels of the patients were nonsense. This was repeated 100 times. Of 100 permutation tests, none of the AUCs were better than of the value 0.692 based on the original un-permuted data. This supports our findings that the predictor performance is not due to random chance—i.e., the chance of the null hypothesis (that we would see this 0.692 AUC performance, by chance alone) is *p* < 0.01.

### 4.2. Comparison of the MS-Based Metabolomics Test with NMR-Based Test

The concentration values of 685 samples measured by LC-MS were compared with the NMR quantifications using Passing and Bablok regression [26,27]. The correlation plots between MS quantifications and NMR quantifications for each of the three metabolites are shown in Figures S3–S5 (Supplemental Data). For all three metabolites, there was a strong positive correlation of MS data with the NMR data (R > 0.8, *P* < 0.01). The regression line equation for ascorbic acid is y = 2.50 + 1.12x; 95% CI for intercept 2.50 to 2.50 and a slope of 1.06 to 1.19 indicated by the small constant and small proportional difference. The regression line equation for carnitine is y = 1.73 + 0.99x; 95% CI for intercept 0.77 to 2.50 and a slope of 0.96 to 1.02 indicated by the small constant and no proportional difference. The regression line equation for succinic acid is y = 4.17 + 1.32x; 95% CI for intercept 2.72 to 5.33 and a slope of 1.26 to 1.38, indicated by the small constant and small proportional difference. For all three metabolites, within the 95% CI, the two methods were not identical, however the values measured from both methods were comparable. For the test performance comparison, a NMR predictor was also built and evaluated using the same analysis workflow as the MS predictor. The AUC of the NMR test is 0.670 which is slightly lower than the AUC of MS based test at 0.692. This might be due to the fact MS is more sensitive in the lower concentration range for these three metabolites.

### 4.3. Comparison of the MS-Based Urine Metabolomics Test with Commercially Available Fecal-Based Tests

The diagnostic accuracies of our developed MS-based test for colonic adenomatous polyps were compared with the three fecal-based (one fecal-guaiac and two fecal-immune) tests. Figure 2a,b show how the performances of these three fecal-based tests compared to the MS-based urine test. Since none of the fecal tests (which measure only a single marker) have an adjustable threshold, each test corresponds to a point in the ROC space. All three fecal tests lie on or below our urine-based predictor’s ROC curve, which indicates that the MS-based urine test always outperforms the fecal tests. The sensitivity, specificity, positive predictive value (PPV) and negative predictive value (NPV) for each test for adenomatous polyp detection on the same training set and testing set are calculated and summarized in Table 3. The overall sensitivities for polyp detection on the total 685 samples by Fecal Guaiac HemII^®^, Fecal Immune ICT^®^, and Fecal Immune MagSt^®^ are 2.6%, 13.2%, and 17.6%, with specificities of 99.0%, 97.1%, and 94.2%, respectively. Although these tests are currently used to screen for colonoscopies, they focus on colon cancer detection, not polyp detection. All three fecal-based tests offer high specificity for polyps but a very low sensitivity (<18%) which makes their use for polyp detection and early cancer screening highly questionable. For the MS-based urine test, a sensitivity of 43.1% and a specificity of 91.3% was achieved for adenomatous polyp detection. At this threshold, the MS-based urine demonstrates a much higher sensitivity (43.1%) compared to all three fecal based tests (sensitivities < 18%), while maintaining the high specificity. For a population-based CRC early screening tool, a highly sensitive test may be demanded. While at another threshold, a sensitivity of 82.4% and a specificity of 36.0% was achieved for adenomatous polyp detection for the MS-based urine test. The MS-based urine metabolomic test designed for adenomatous polyp detection at high sensitivity would serve as a better population based screening tool for CRC.

## 5. Discussion

The metabolites and clinical features used in the algorithm for the MS-based urine test are summarized in Table 4. Correlations were calculated by encoding those patients who are likely to have adenomatous polyps and require colonoscopy as “1” and those that do not as “0”. Higher concentrations of the 3 metabolites were inversely correlated with the presence of adenomatous polyps (e.g., lower concentrations of each metabolite indicate the patient is more likely to have adenomatous polyps and require colonoscopy). Since the sex feature was encoded with males as being 1, and females 0, a patient being male is positively correlated with the presence of adenomatous polyps and the need for colonoscopy. Age is also positively correlated, with older patients more likely to have adenomatous polyps present and need a colonoscopy. Finally, the fact that a patient smokes is directly correlated with the need for colonoscopy (i.e., smokers are more likely to develop polyps). These correlation findings of clinical features with the adenomatous polyps align with many previous findings for correlation of these clinical features with CRC [28,29]. Although none of the correlations associated with each feature has a large absolute value, the linear combination of multiple features can yield a strong correlation for adenomatous polyps.

Carnitine (HMDB00062) and succinic acid (HMDB00254) have previously been found to be associated with colorectal cancer [30]. Additionally, ascorbic acid (HMDB00044) has been linked to other kinds of cancers [31]. Carnitine is necessary for fatty acid oxidation and transporting fatty acids from the cytosol to the mitochondria, where it is broken down via the citric acid cycle to release energy. In humans, most of the carnitine in the body comes from dietary sources such as red meat and dairy products. About 25% of carnitine is synthesized in the liver, kidney, and brain from the amino acids lysine and methionine [32]. Interestingly, the final step of carnitine synthesis pathway occurs as 4-trimethylammoniobutanoic acid is transformed into carnitine via the enzyme gamma-butyrobetaine dioxygenase where ascorbic acid acts as coenzyme and succinic acid gets produced. Succinic acid is a dicarboxylic acid. The anion, succinate, is a component of the citric acid cycle capable of donating electrons to the electron transfer chain. Oxidizing succinate links succinate dehydrogenase (SDH) to the fast-cycling Krebs cycle portion where it participates in the breakdown of acetyl-CoA throughout the whole Krebs cycle [33]. Succinic acid is also involved in ketone body metabolism and butyrate metabolism. Succinic acid is produced in carnitine synthesis, oxidation of branched chain fatty acids, glutamate metabolism, citric acid cycle, arginine and proline metabolism, and valine, leucine and isoleucine degradation [34]. Ascorbic acid is considered an antioxidant and functions as a reducing agent and a coenzyme in several metabolic pathways. The biologically active form of ascorbic acid is vitamin C which cannot be produced in the human body and must be obtained in food. Ascorbic acid is an electron donor for enzymes involved in collagen hydroxylation, biosynthesis of carnitine and norepinephrine, tyrosine metabolism, and the amidation of peptide hormones [12].

This work has leveraged urine metabolomics and patient medical histories to predict whether a person has adenomatous polyps and so should receive a colonoscopy. We have successfully transferred a NMR-based assay to a MS-based assay with improved performance. Mass spectrometers are commonly used in clinical environments which makes this MS-based test easy to adopt for clinical use. In fact, Metabolomic Technologies Inc. has launched the MS-based urine test, PolypDx™, in the USA through collaborating with the Clinical Laboratory Improvement Amendments (CLIA) certified laboratories. This MS-based urine test performs better than fecal-based tests, and offers additional advantages with regard to patient compliance, ease of sample collection, and performance tunability. It is also one of the first true metabolomic (i.e., multi-metabolites) tests to be brought into the clinical environment.

## Figures and Tables

**Table 1:** Participant characteristics of the 685 participants for this study.

Label	Colonoscopy Results	Age	Sex	Smoker
**Normal**	Normal (*n* = 446)	μ = 56.1	F = 308	Yes = 50
***n***** = 530**	Hyperplastic (*n* =84)	σ = 8.2	M = 222	Ex-Smoker = 12
				No = 449
				Unknown = 19
**Polyp**	Adenoma (*n* = 154)	μ = 59.9	F = 60	Yes = 26
***n***** = 155**	CRC (*n* = 1)	σ = 7.4	M = 95	Ex-Smoker = 4
				No = 119
				Unknown = 6

**Table 2:** Top 10 *p*-values for metabolites in NMR data using the Wilcoxon signed-rank test.

*P*-Value	Metabolite
0.0059	Succinic acid
0.0100	Ascorbic acid
0.0280	Carnitine
0.0595	Creatine
0.0739	Citric acid
0.0861	Methylamine
0.0945	Pantothenic acid
0.1198	Fumaric acid
0.1346	1-Methylnicotinamide
0.1703	Trigonelline

**Table 3:** The performance of MS-based urine tests for the detection of adenomatous polyp on both training set and testing set, along with the performance of three fecal based tests. When picking a threshold on the training set, the performance on the testing set with the same threshold produces similar performance.

	Training Set				Testing Set			
Threshold Criteria	Sensitivity	Specificity	PPV	NPV	Sensitivity	Specificity	PPV	NPV
**Urine tests**								
Sens = 90% (95% CI *)	90.3% (84.6–96.0%)	20.9% (16.7–25.1%)	24.9%	88.0%	92.2% (84.8–99.5%)	19.2% (13.3–25.1%)	25.3%	89.2%
Sens = 80% (95% CI)	79.6% (71.8–87.4%)	42.1% (36.9–47.2%)	28.6%	87.7%	**82.4%** (71.9–92.8%)	**36.0%** (28.9–43.2%)	27.6%	87.3%
Sens = 70% (95% CI)	69.9% (61.0–78.8%)	59.0% (53.9–64.2%)	33.2%	87.1%	66.7% (53.7–79.6%)	55.2% (47.8–62.7%)	30.6%	84.8%
Spec = 70% (95% CI)	59.2% (49.7–68.7%)	70.1% (65.3–74.8%)	36.5%	85.5%	56.9% (43.3–70.5%)	70.9% (64.1–77.4%)	35.4%	84.7%
Spec = 80% (95% CI)	46.6% (37.2–56.2%)	80.0% (75.8–84.1%)	40.3%	83.7%	49.0% (35.3–62.7%)	80.8% (74.9–86.7%)	43.1%	84.2%
Spec = 90% (95% CI)	31.1% (22.1–40.0%)	88.1% (84.8–91.5%)	43.2%	81.4%	**43.1%** (29.5–56.7%)	**91.3%** (87.1–95.5%)	59.5%	84.4%
**Fecal Tests**								
Guaiac HemII	2.0%	98.8%	33.3%	77.5%	3.8%	99.4%	66.7%	77.1%
Immune ICT	10.9%	97.1%	52.4%	78.7%	17.6%	97.0%	64.3%	79.6%
Immune MagSt	15.8%	95.4%	50.0%	79.5%	21.2%	91.7%	44.0%	79.1%

* CI: confidence intervals. They were estimated based on based on binomial distribution.

**Table 4:** Further Information about features used in the algorithm for MS-based urine test.

Feature	PubChem CID	HMDB	Correlation
**Smoker**	N/A	N/A	0.09
**Age**	N/A	N/A	0.13
**Sex**	N/A	N/A	0.17
**Succinic Acid**	1110	HMDB00254	−0.16
**Ascorbic Acid**	54670067	HMDB00044	−0.15
**Carnitine**	2724480	HMDB00062	−0.13

## Supplementary Materials

The following are available online at www.mdpi.com/2218-1989/7/3/32/s1. Figure S1. A representative LCMS of Calibrant 6; Figure S2. A representative plate map. LCMS sequence runs vertically; Figure S3. Passing and Bablok regression analyses of MS-quantified on NMR-quantified data for Succinic acid, N = 685; concentration range 0–362 μmol/L; Pearson correlation coefficient r = 0.862, P < 0.0001; Figure S4. Passing and Bablok regression analyses of MS-quantified on NMR-quantified data for Ascorbic acid, N = 685; concentration range 0–13,368 μmol/L; Pearson correlation coefficient r = 0.800, P < 0.0001; Figure S5. Passing and Bablok regression analyses of MS-quantified on NMR-quantified data for Carnitine, N = 685; concentration range 0–948 μmol/L; Pearson correlation coefficient r = 0.921, P < 0.0001; Table S1. Optimized MS parameters for each compound. MRM pair 1 is used for quantitation and MRM pair 2 is for qualification; Table S2. Extraction recoveries and accuracies for each metabolite; Table S3. CV% of QC samples for each metabolite within each plate.
