# Supplementary material for: Development and Validation of a High-Throughput Mass Spectrometry Based Urine Metabolomic Test for the Detection of Colonic Adenomatous Polyps
Source: Metabolites. 2017 Jun 22;7(3):32. doi: 10.3390/metabo7030032 (PMC5618317; doi:10.3390/metabo7030032)
Supplement: Supplementary file 1 [file metabolites-07-00032-s001.pdf]

Supplementary Materials: Development and validation of a high-throughput mass spectrometry based urine metabolomic test for colorectal cancer screening

Lu Deng, David Chang, Rae R. Foshaug, Roman Eisner, Victor K. Tso, David S. Wishart, Richard N. Fedorak

Table S1. Optimized MS parameters for each compound. MRM pair 1 is used for quantitation and MRM pair 2 is for qualification.

Compound	Polarity	Q1	Q3	DP	CE	CXP
Succinic acid 1	-	117.0	73.0	-40	-16	-1
Succinic acid 2	-	117.0	55.1	-40	-22	-7
Succinic acid-D4	-	121.0	77.0	-40	-16	-1
Ascorbic acid 1	-	175.0	114.9	-45	-18	-7
Ascorbic acid 2	-	175.0	86.8	-45	-28	-13
Ascorbic acid-^{13}C	-	176.0	116.0	-45	-18	-7
Carnitine 1	+	162.1	103.1	51	25	6
Carnitine 2	+	162.1	43.2	51	47	6
Carnitine-D9	+	171.0	103.0	51	25	6

Table S2. Extraction recoveries and accuracies for each metabolite.

Metabolite	Recovery (%)[a]	Accuracy (%)[b]
Succinic acid	101.0	110.2
Ascorbic acid	93.8	98.7
Carnitine	93.1	102.7

a. Recovery (%) = (Response (spiked sample))/(Response (post-spiked sample)) x 100

b. Accuracy (%) = (spiked sample-upspiked sample)/(spiked amount) x 100

Table S3. CV% of QC samples for each metabolite within each plate.

	Succinic acid		Ascorbic acid		Carnitine	
	Average concentration (μM)	CV%	Average concentration (μM)	CV%	Average concentration (μM)	CV%
**Kit 1**	21.1	14.7%	125.2	8.6%	45.5	9.8%
**Kit 2**	23.0	5.9%	121.8	8.3%	49.2	10.4%
**Kit 3**	25.7	16.7%	109.0	10.2%	46.2	8.4%
**Kit 4**	25.3	5.3%	119.6	4.1%	49.5	5.5%
**Kit 5**	25.8	10.3%	135.0	7.7%	47.7	4.3%
**Kit 6**	27.7	13.6%	107.0	4.1%	46.7	6.4%
**Kit 7**	11.4	13.1%	104.7	14.0%	25.9	10.9%
**Kit 8**	14.0	11.7%	123.0	4.5%	35.0	6.0%
**Kit 9**	17.4	12.5%	114.6	9.1%	40.3	11.9%
**overall**	21.1	10.0%	116.7	6.8%	42.6	7.6%

coefficient of variation (CV%) = (standard deviation)/(mean value)

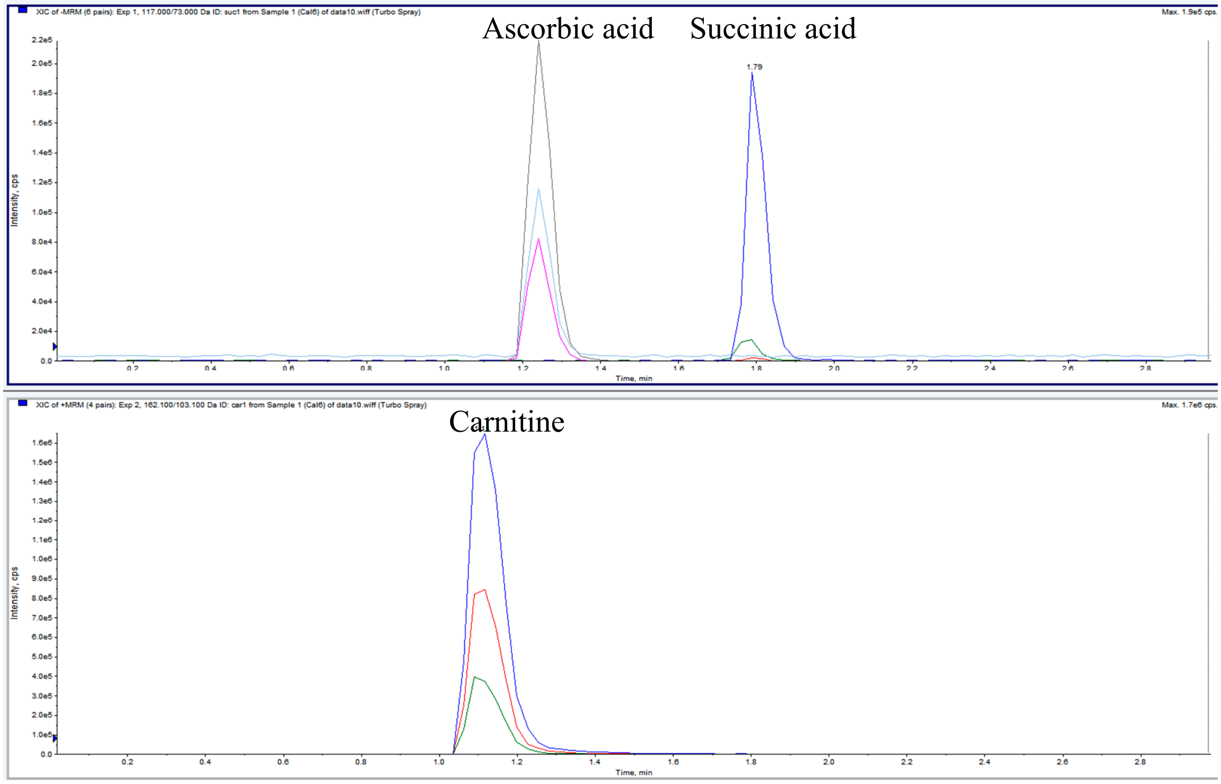

Figure S1. A representative LCMS of Calibrant 6.

	1	2	3	4	5	6	7	8	9	10	11	12
A	Blank	Cal7	Urine6	Urine14	Urine21	Urine29	Urine36	Urine44	Urine51	Urine59	Urine66	Urine74
B	ISTD	Cal8	Urine7	Urine15	Urine22	Urine30	Urine37	Urine45	Urine52	Urine60	Urine67	Urine75
C	Cal1	Urine1	Urine8	Urine16	Urine23	Urine31	Urine38	Urine46	Urine53	Urine61	Urine68	Urine76
D	Cal2	Urine2	Urine9	Urine17	Urine24	Urine32	Urine39	Urine47	Urine54	Urine62	Urine69	Urine77
E	Cal3	Urine3	Urine10	Urine18	Urine25	Urine33	Urine40	Urine48	Urine55	Urine63	Urine70	Urine78
F	Cal4	Urine4	Urine11	Urine19	Urine26	Urine34	Urine41	Urine49	Urine56	Urine64	Urine71	Urine79
G	Cal5	Urine5	Urine12	Urine20	Urine27	Urine35	Urine42	Urine50	Urine57	Urine65	Urine72	Urine80
H	Cal6	QC	Urine13	QC	Urine28	QC	Urine43	QC	Urine58	QC	Urine73	QC

Figure S2. A representative plate map. LCMS sequence runs vertically.

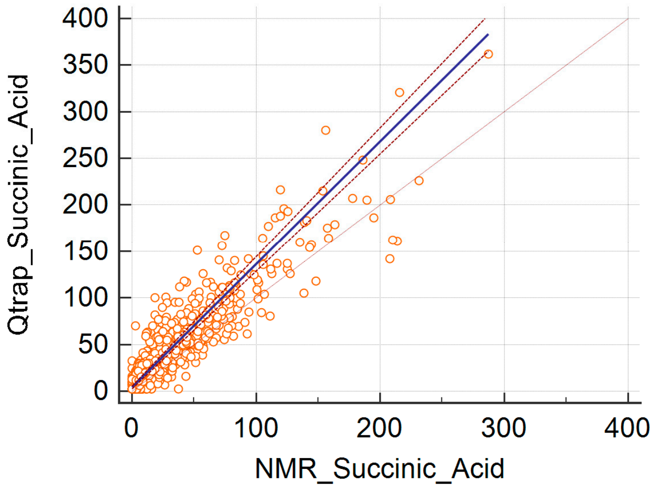

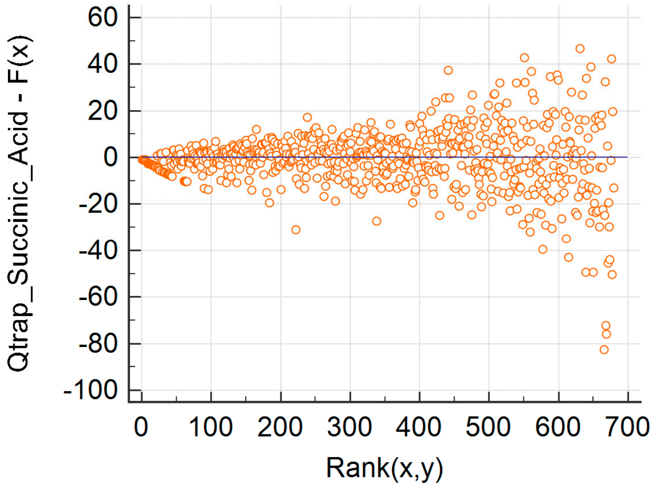

Figure S3. Passing and Bablok regression analyses of MS-quantified on NMR-quantified data for Succinic acid, N = 685; concentration range 0-362 μmol/L; Pearson correlation coefficient r = 0.862, P < 0.0001. (**A**) Scatter diagram with regression line and confidence bands for regression line. Identity line is dashed. Regression line equation: y = 4.17 + 1.32 x; 95% CI for intercept 2.72 to 5.33 and for slope 1.26 to 1.38 indicated small constant and small proportional difference. Cusum test for linearity indicates significant deviation from linearity (P<0.01). (**B**) Residual plot presents distribution of difference around fitted regression line.

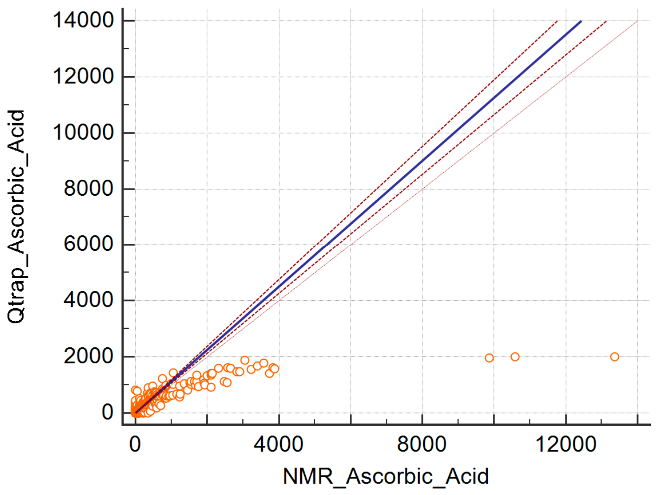

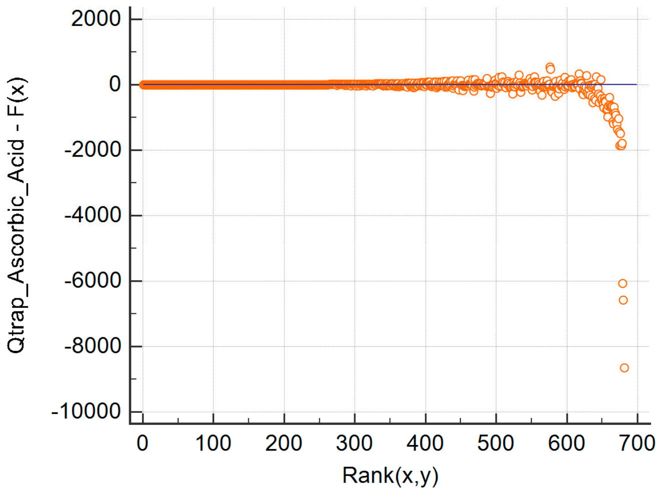

Figure S4. Passing and Bablok regression analyses of MS-quantified on NMR-quantified data for Ascorbic acid, N = 685; concentration range 0-13368 μmol/L; Pearson correlation coefficient r = 0.800, P < 0.0001. (**A**) Scatter diagram with regression line and confidence bands for regression line. Identity line is dashed. Regression line equation: y = 2.50 + 1.12 x; 95% CI for intercept 2.50 to 2.50 and for slope 1.06 to 1.19 indicated small constant and small proportional difference. Cusum test for linearity indicates significant deviation from linearity (P<0.01). (**B**) Residual plot presents distribution of difference around fitted regression line.

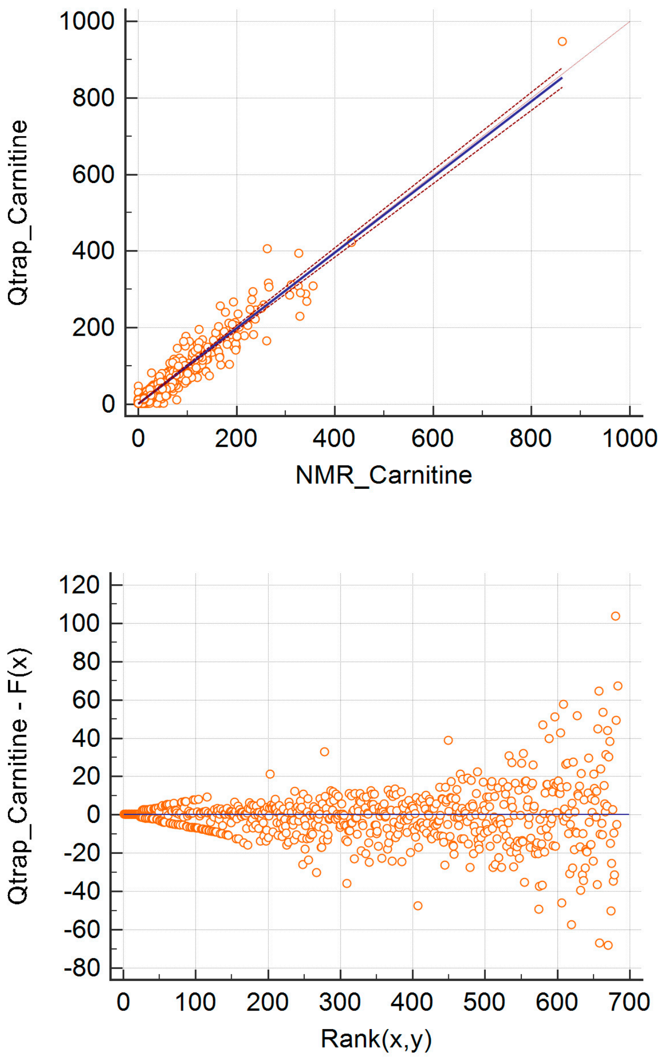

Figure S5. Passing and Bablok regression analyses of MS-quantified on NMR-quantified data for Carnitine, N = 685; concentration range 0-948 µmol/L; Pearson correlation coefficient r = 0.921, P < 0.0001. (**A**) Scatter diagram with regression line and confidence bands for regression line. Identity line is dashed. Regression line equation: y = 1.73 + 0.99 x; 95% CI for intercept 0.77 to 2.50 and for slope 0.96 to 1.02 indicated small constant and small proportional difference. Cusum test for linearity indicates significant deviation from linearity (P=0.04). (**B**) Residual plot presents distribution of difference around fitted regression line.
